# Pulmonary Tuberculosis

**Published:** 1894-04-21

**Authors:** 


### Medical Progress.

### PULMONARY TUBERCULOSIS (*continued.*).

*Climatic Treatment.* — Professor Jaccoud,[1] an acknowledged authority on the question of climatic treatment of phthisis, in a valuable article gives us much useful information on the point, the result of thirty years of investigation and experience.

Who are those among the patients affected with pulmonary tuberculosis to whom sojourn in higher altitude resorts ought to be prescribed?

First he draws attention to the fact that the intensity of the special characteristics of the various so-called winter resorts of Switzerland is in direct ratio to their altitude, and the altitude increases regularly from those in the west to those in the east of Switzerland, and may be classified thus: Lower western resort—Leysin, 4,757 feet. Middle eastern resorts—Wiesen, 4,770 feet; Davos-Platz, 5,105 feet; Clavadel, 5,459 feet. Extreme eastern resorts—Samaden, 5,670 feet; St. Moritz-Kulm, 6,090 feet; Upper Arosa, 6,208 feet.

The fundamental character of these climates is briefly as follows: (1) Great rarefaction of the air; (2) cold winters, less subject to variations of the temperature than the summer; (3) more or less protracted season of solid snow; (4) dry and pure air; (5) no wind ordinarily in winter time; (6) no lasting fogs or clouds; (7) intense light; (8) powerful solar radiation. These climatical conditions of high altitudes produce upon the organism, in all seasons, a series of effects, which all fulfil the fundamental requisites of a treatment for phthisis.

Of these effects some are general, determined by the powerful tonic action of high altitudes, namely, increase of appetite and digestive capacity, augmentation of muscular force and power of motion, stimulation of the nervous system.

The other effects of special nature, the direct result of the rarefaction of the air, are the following: Numerical increase of the red blood corpuscles; quantitative increase of hæmoglobin; increased capacity of the blood for absorbing oxygen; increased activity of the nutritive process and organic exchanges, and hence an increase in the quantity of carbonic acid in the air exposed; permanent and unconscious increase of expansion of the lungs and thorax in inspiration: increased activity of the cardio-pulmonary circulation; diminution of the amount of blood in the lungs; and lastly, increased pulmonary evaporation, resulting in proportional refrigeration of the lungs and tendency to dessication.

While the more general effects of tonicity and stimulation subsist during the whole year, the effect of excitation is most marked in the winter when the temperature is lowest.

What are the contra-indications to this therapeutic use of high altitudes?

1. Those conditions which are independent of the tubercular disease, viz., the nervous temperament approaching a state of permanent erethism or excitement; diseases of the heart and circulatory system; disposition to the hæmorrhagic diathesis; extensive pulmonary emphysema and asthma, the last at any rate when the paroxysms become more frequent and severe as soon as the patient ascends ever so little above the level where he lives habitually.

2. Those due to the phthisical condition itself, viz., the pneumonic form of phthisis; when existing in persons whose reaction to high altitudes is very marked; acute manifestations of the disease; in all febrile cases where the fever is continuous; or in those presenting the remittent type. In respect of intermittent fever with vesperal exacerbations, this alone does not constitute a contra-indication.

A previous attack of hæmoptysis, even if repeated on several occasions, does not furnish an absolute contra-indication, provided the patient does not show the kind of reaction designated erethism. The extent of the pulmonary lesions is an important consideration, more so than the degree of the lesion. When very extensive, the patient, from want of sufficient lung tissue available for hæmatosis, would be a prey to permanent dyspnœa.

He summarises the indications for the resort to high altitudes by saying that they are suitable for consumptives with sluggish or indifferent reaction, when the disease is of a chronic nature, and has developed without marked or frequent acute periods, the lesions being circumscribed, and when there is no sign of a serious complication in either the larynx, intestine, or kidneys, nor of the patient's approaching the consumptive stage.

It is always best to acclimatise the patient in the spring or summer, and not to send them to the high altitudes in the winter when the factors producing great reaction are most marked; and the Professor urges that it is essential that the residence at these high elevations should be prolonged, and not simply a matter of a few winter months.

A melancholy interest attaches to Dr. Keating's[2] article on "Climate as one Element of Cure," the last contribution penned by this authority, and as the editor of the *International Medical Magazine* tells us, was to have been used as the commencement of a work on climatology. He emphasises the importance of prescribing climatic resorts intelligently after due consideration of the case in all its details, including the financial, mental, and social conditions of the patient. There is no one particular climate nor one particular resort which is suitable for every case. . . . It is well enough to say to a patient, "Go to Colorado, to New Mexico, to Southern California, to Arizona," but should he not be told specifically, after a thorough understanding of the case, to what objective point he should go to find all the advantages which should enable him to benefit by a residence at that particular point?

Like Jaccond, Keating considers it necessary to lay stress on prolonged residence. "For one to thoroughly secure all the advantages of a climate one must become a resident and not a mere sojourner. This, unfortunately, is not sufficiently understood."

Denver and Davos compared by Carl Ruedi brings into relief some important points that have to be considered with increasing frequency. But though both these places are at exactly the same altitude, this being really the only point in which they resemble each other, the difficulty in making a fair comparison is very great. The title of the article is "A Comparison of the Winter Health Resorts in the Alps with Some Places in the Rocky Mountains," for the abundant growth of apples, pears, chestnuts, water-melons, and wheat at Hygiene, only 200 feet lower than Davos, were so strikingly in contrast with the summer of Davos itself that Ruedi was led to look out for places in Colorado which have the same character and fauna, &c., as Davos and St. Moritz, and now he does not hesitate to say that the Eastern slope of the Rocky Mountains offers as good climatic conditions as the Eastern resorts, or even better.

As the result of his investigations, and of reports prepared at his suggestion, the author forms the following comparison between the European and Colorado resorts:—

1. The barometrical pressure shows decidedly less variation in Colorado.

2. Humidity is one of the most striking points of differentiation. Not only is the absolute rainfall considerably greater in the Swiss mountains, but the number of days on which precipitation occurs is much higher.

3. The sunshine is much more constant in the Rocky Mountain resorts.

4. The temperature of the Rocky Mountain resorts, *e.g.*, Ester Park, is remarkably even.

5. A point of difference which is very striking to the newcomer to Colorado is the increased electricity. He cannot understand why electricity has not been studied more climatologically, because the difficulties can be overcome, and there is no doubt that this factor is of equal importance with temperature and humidity. He reminds us of one observation on Mount Lowry, near Paris, where the bacteriologists found their culture of germs and fungi did not grow during the time that the air was positively electric, but they grew well while the air was negatively electric.

6. The wind. Colorado enjoys the name of being a windy place, and so it is in many quarters, but such spots as Denver and Colorado Springs are no criterion for the real mountain places in the centre of the Rocky Mountains themselves. Mr. Killby, writing to him from Ester Park, said, "There is no relaxing or moist wind corresponding to the Föhu which prevails in Davos-Platz; the prevailing winds are from the west to south-west, but not, as a rule, such as to prevent invalids from exercising in the open air." The author states that non-windy Davos often suffers from want of it because the smoke hovers over the valley, and it is this fault tending to increase with cheapness of coal at Davos and the increased house accommodation which affords such good prospects for St. Moritz and Arosa.

Denver very closely resembles the Lake of Zurich, with the exception of the altitude and the dryness of the air; Colorado Springs resembles the Valley of the Rhine at Ragatz and Mazenfeld, but is always dry and has also a higher altitude, and for the real mountain health resorts nearly equal to Davos and St. Moritz. He would propose Ester Park at an elevation of 6,940 feet.

*Remedies.—Cod-liver Oil*; *its Properties*, formed the subject of discussion at a recent meeting of the Therapeutical Society, Paris, being introduced by Patein,[3] who said that some authorities claim that cod-liver oil is a specific remedy, owing its efficacy to certain metalloids and alkaloids, of which Gauthier especially has made a thorough study. Others say that cod-liver oil is an excellent food substance, rich in fatty elements, capable of being easily assimilated. A third opinion, of eclectic nature, is to the effect that cod-liver oil possesses both nutritive and remedial qualities at the same time. Its physical, chemical, and therapeutical properties, indeed, depend largely upon the manner of its extraction and the time when such extraction takes place. Differences of this order account for the three varieties current in the trade, viz., white or pale yellow, brownish yellow, and dark brown cod-liver oil. Pharmacists and clinicians are agreed in considering as the best the brownish yellow oil, which has the colour of Madeira wine. Cod-liver oil is more easily assimilated than other oils, because it contains a certain quantity of the elements of bile, which facilitate its absorption by the intestinal villi.

The presence of iodine and phosphorus, and the existence of alkaloids discovered by Gauthier and Mourgues, suffice to explain its tonic, reconstituent, thermogenic, and dynamic properties. The therapeutical applications of cod-liver oil are varied and numerous, but it is principally in the treatment of tuberculosis and rachitis that it has proved most efficacious. All the different forms of tuberculosis, however, are not in the same degree amenable to treatment by cod-liver oil; and its use is, therefore, specially indicated in the torpid, scrofulous forms of this disease, or at the beginning of the slow form of chronic tuberculosis. On the other hand, it seems to be contraindicated in the febrile, hæmoptic forms. At the cachectic stage its efficacy is more than doubtful. In rachitis, cod-liver oil constitutes, so to speak, the specific treatment. In chronic rheumatism, contrary to old and firmly established belief, it appears to have, in reality, no effect whatever.

By adding the essential oil of bitter almonds or of eucalyptus, the odour of the cod-liver oil may be disguised; and the administration is greatly facilitated by taking the precaution to moisten the glass, so as to prevent the oil adhering to the sides. Peptonisation is absolutely useless, because the quantity of albuminoid substances which cod-liver oil actually contains is very small indeed, hardly 1·5 per cent. As regards pancreatinisation, Patein does not believe that we know enough of the real properties of the artificial pancreatic ferments to be able to judge of their influence. The gastric juice in no way alters the cod-liver oil, which is acid, and only affected by the intestinal digestion.

An emulsion, called hydroleine, having the following composition has been introduced recently: 120 drops contain, pure Norwegian cod-liver oil, 80 m. (drops); distilled water, 35 m.; soluble pancreatine, 5 grains; soda, ½ grain; salicylic acid, ¼ grain.

[1]Med. Week, Mar. 9, 1894. [2] International Medical Mag., Jan., 1894. [3] Med. Week., Mar. 9, 1894.

